# Development a novel multiepitope DNA vaccine against human SARS coronavirus-2: an immunoinformatic designing study

**DOI:** 10.55730/1300-0152.2615

**Published:** 2022-06-23

**Authors:** Afshin Samimi NEMATI, Sako MIRZAIE, Mohammad Reza MASOUMIAN, Fatemeh SHEIKHI, Mostafa JAMALAN

**Affiliations:** 1 Department of Biochemistry, Abadan University of Medical Sciences, Abadan, Iran; 2 Advanced Pharmaceutics and Drug Delivery Laboratory, Leslie L. Dan Faculty of Pharmacy, University of Toronto, Toronto, Canada

**Keywords:** DNA vaccine, reverse vaccinology, immunoinformatic, COVID-19, SARS-CoV-2

## Abstract

Human SARS coronavirus 2 (SARS-CoV-2) causes the current global COVID-19 pandemic. The production of an efficient vaccine against COVID-19 is under heavy investigation. In this study, we have designed a novel multiepitope DNA vaccine against SARS-CoV-2 using reverse vaccinology and DNA vaccine approaches. Applying these strategies led to reduce the time and costs of vaccine development and also improve the immune protective characteristics of the vaccine. For this purpose, epitopes of nucleocapsid, membrane glycoprotein, and ORF8 proteins of SARS-CoV-2 chose as targets for B and T-cell receptors. Accordingly, DNA sequences of selected epitopes have optimized for protein expression in the eukaryotic system. To this end, the Kozak and tissue plasminogen activator sequences were added into the epitope sequences for proper protein expression and secretion, respectively. Furthermore, interleukin-2 and beta-defensin 1 preproprotein sequences were incorporated to the designed DNA vaccine as an adjuvant. Modeling and refinement of fused protein composed of SARS-CoV-2 multiepitope antigens (fuspMA) have performed based on homology modeling of orthologous peptides, then constructed 3D model of fuspMA was more investigated during 50 ns of molecular dynamics simulation. Further bioinformatics predictions demonstrated that fuspMA is a stable protein with acceptable antigenic features and no allergenicity or toxicity characteristics. Finally, the affinity of fuspMA to the MHC I and II and TLRs molecules validated by the molecular docking procedure. In conclusion, it seems the designed multiepitope DNA vaccine could have a chance to be introduced as an efficient vaccine against COVID-19 after more in vivo evaluations.

## 1. Introduction

The novel human severe acute respiratory syndrome coronavirus 2 (SARS-CoV-2) that causes coronavirus disease 2019 (COVID-19) is a positive-sense single-stranded RNA (+SS-RNA) virus (Park, 2020; Velavan and Meyer, 2020). SARS-CoV-2 belonged to the Coronaviridae family and genus of Betacoronavirus (Helmy et al., 2020). COVID-19 was first observed as a respiratory syndrome disease at the end of 2019 in Wuhan city of China and is responsible for significant mortality and morbidity in the human population in the current worldwide pandemic (Wu et al., 2020). Previously, various outbreaks of infections caused by bacteria, viruses, and other pathogens have been reported. In a recent one, the previous outbreak of the SARS coronavirus in 2002–2004 caused several deaths (Ksiazek et al., 2003; Anderson et al., 2004). Nowadays, COVID-19 spread around the world and became a crucial health problem with various mutations leading to create different variants of viruses such as Deltacoronavirus, Alphacoronavirus, Gammacoronavirus and the case which recently spread along the globe, Omicron coronavirus (Wu et al., 2020; Salcedo et al., 2022).

**Figure 1:**
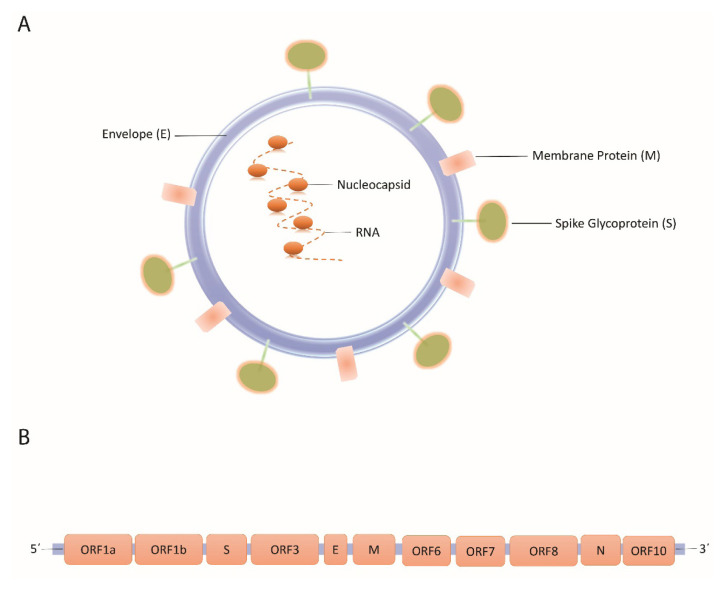
(A) Schematic presentation of structure of SARS-CoV-2. (B) Sequences of genes in genome of SARS-CoV-2 (Mohammad et al., 2020). In current project, epitopes from the nucleocapsid proteins, membrane glycoprotein, and ORF8 protein selected and evaluated for deigning an efficient DNA vaccine.

Efficient vaccination is the most relevant approach to prevent the contagion of an infection and subsequent challenges. Various procedures have been developed for vaccine design and production since attenuated-infectious agents until the novel DNA vaccines. The development of multiepitope DNA vaccines by using the reverse vaccinology (RV) approach is a novel and innovative strategy for vaccine production (Li and Petrovsky, 2016; Enayatkhani et al., 2020). The RV approach was first invented by Rappouli for the B serotype of *Neisseria meningococcus* and after that, it has been used in a variety of vaccine design projects (Rappuoli, 2001; Rappuoli et al., 2016). Currently, improvement in immunoinformatic by simulation and prediction of B and T-cell epitopes in available data banks have contributed to the development of DNA vaccines based on the RV method (Dhanda et al., 2019). Today, this method has been attended by researchers because of its advantages like requiring short time for design and production of the candidate vaccine and induction of an adequate immune protective result. Despite the traditional vaccine development methods, designing a DNA vaccine based on the RV approach has noticeable advantages. To produce a multiepitope-based vaccine in its peptide form, the DNA sequence of the related protein should be synthesized, be expressed in conventional hosts, and finally expressed antigens should be purified and prepared for delivery to individuals. Therefore, peptide-based vaccine development and production could be expensive and time-consuming on a large scale because of indicated steps. Despite of peptide-based vaccines, DNA vaccines as a new generation of vaccines containing just a DNA sequence of some selected antigens to induce immunity against the indicated pathogen. Using DNA vaccines could reduce costs and time for the vaccine invention and production processes simultaneously, while it has also its challenges (Heppell and Davis, 2000). Nevertheless, there are different reports about the design and production of DNA vaccines, to our acknowledge, none of them were approved for human beings until the covid-19 pandemic appearance (Silveira et al., 2021). In the current work, we have designed a DNA vaccine by combining the sequences of the most potent epitopes of SARS-CoV-2. This kind of designed DNA vaccine can reduce the time and costs of the prepared vaccine in comparison to similar peptide-based vaccines. This strategy could increase the number of the presented antigens to immune cells in comparison to one-epitope DNA vaccines. According to the importance of surface proteins that could induce a strong and effective immune response, the nucleocapsid protein and membrane glycoprotein were selected for epitope prediction (Figure 1). Furthermore, the ORF8 protein with a high rate of antigenicity was selected for vaccine designing (Figure 1) (Kumar et al., 2013). To enhance the immunogenicity of the proposed vaccine, protein adjuvants were added to the designed peptide sequence. For this reason, the amino acid sequence of beta-defensin 1 preproprotein and interleukin-2 added into the proposed vaccine sequence (Barouch et al., 2004). Moreover, to design an effective DNA vaccine some features such as necessary elements for gene expression and protein production, proper vectors and promoters, and suitable delivery systems must be considered to achieve an appropriate vaccine (Manam et al., 2000). To this aim, the Kozak sequence was used in the initial part of the nucleic sequence of predicted epitopes and sequence of tissue plasminogen activator (TPA) was used as a signal peptide element (Garmory et al., 2003). The TPA sequence acts as a signal peptide that causes to secrete the desired protein into the extracellular environment of the eukaryotic aimed cells. This process could lead to a more proper exposition of the proposed sequence of vaccines within the immune system to make an effective immune response. The final chimeric-designed DNA sequence can clone in a suitable vector for further investigations. In the end, the cloning of the final sequence containing the Kozak sequence, TPA, TEV protease cleavage site into the DNA expression pVax1 vector simulated by SnapGene software (Xu et al., 2017).

## 2. Materials and methods

### 2.1. Primary data collection

The first step of this study began by retrieving related protein sequences of human SARS coronavirus 2. Therefore, sequences of structural proteins of the human coronavirus 2 were obtained in FASTA format from the NCBI database. In a similar way, interleukin-2 (GenBank: AAB46883.1) and beta-defensin 1 preproprotein (NP_005209.1) sequences retrieved in FASTA format from NCBI database (https://www.ncbi.nlm.nih.gov/). Also, the three-dimensional structure and amino acid sequence of human TLR3, TLR4, MHC I, and MHC II in PDB format were obtained from protein data bank (https://www.rcsb.org/) (Berman et al., 2007).

### 2.2. Choosing targeted proteins for designing chimeric DNA vaccine

After primary analysis, the nucleocapsid protein (GenBank: QHO62110.1), membrane glycoprotein (GenBank: QHR84452.1), and ORF8 protein (GenBank: QHW06065.1) were selected as target proteins. For this goal, the antigenicity level of considered proteins was evaluated by using the Vaxijen 2.0 server (http://www.ddg-pharmfac.net/vaxijen/VaxiJen/VaxiJen.html ) (Doytchinova and Flower, 2007). In continue, the allergenicity and toxicity of the selected proteins were studied by AllerTOP v. 2.0 (https://www.ddg-pharmfac.net/AllerTOP) (Dimitrov et al., 2014) and Toxin Pred server (https://webs.iiitd.edu.in/raghava/toxinpred/algo.php) (Gupta et al., 2013), respectively.

### 2.3. Epitope prediction of targeted proteins

The immune epitope database (IEDB), which is available on https://www.iedb.org/, is an applicable tool for epitope prediction (Vita et al., 2019). This server contains various tools that are used widely in immunoinformatics studies. In this study, we used this server to predict B and T-cell epitopes. The linear B-cell epitopes have been predicted using available methods and also the MHC I and II related epitopes have been predicted by the T-cell epitope prediction tool of the server. Furthermore, all alleles for MHC I and II molecules were considered in the epitope prediction process to achieve a universal vaccine.

### 2.4. Choosing epitopes with high antigenicity, low allergenicity, and low toxicity to construct a multiepitope DNA vaccine

To design an appropriate vaccine, antigenicity and allergenicity of the selected epitopes are so crucial. High antigenicity levels and lack of allergenicity properties of an epitope could lead to a stronger immune reaction and reduction of allergenic responses (Reese et al., 2007). According to the importance of this point, the epitopes with higher antigenic features and nonallergen ones were chosen from the predicted epitopes. Moreover, for T-cell epitopes selection, epitopes that covered most of HLA alleles were considered. Also, due the fact that some amino acid orders in peptide sequences may cause a toxic function, the toxicity of the selected epitopes sequences was predicted using the Toxin Pred server as mentioned above (Gupta et al., 2013).

### 2.5. Design the amino acid sequence of aimed vaccine

Finally, sequences of selected epitopes should be attached together to make a united peptide sequence that was named fused protein composed of SARS-CoV-2 multiepitope antigens (fuspMA of SARS-CoV-2). To this aim, AAY linkers were used to connect the amino acid sequence of selected epitopes. In the next step, to increase the probability of exposing the immune system and take a stronger immune reaction against the candidate vaccine, a sequence of protein adjuvants was added to the peptide sequence. For this, the amino acid sequence of beta-defensin 1 preproprotein added into the N terminal and interleukin-2 sequence added into the C-terminal of the proposed vaccine sequence using EAAAK linkers.

### 2.6. Determination of physicochemical properties and stability of the designed protein

In this section of the study, the physicochemical properties of the fuspMA of SARS-CoV-2 protein such as half-life, the amino acid compositions, the isoelectric pH (pI), and the native charge in pH 7 were analyzed by using related servers. Innovagen, PepCalc.com–Peptide property calculator (https://pepcalc.com/) and Protparam (http://web.expasy.org/cgi-bin/protparam/) servers (Gasteiger et al., 2005) were used for this aim. In the following, for prediction of protein disorder as a function of redox state and potential of protein binding of fuspMA of SARSCoV-2, IUPred 2a (https://iupred2a.elte.hu/) server was applied (Mészáros et al., 2018). IUPred2A server reported the results in a graph with a threshold of the area under the curve (AUC) values that can range from 0.5 for random predictions to 1 for perfect predictions (Mészáros et al., 2018).

### 2.7. Prediction of secondary and 3D structure of fuspMA of SARS-CoV-2

The structure of a protein is an important issue for its expression, function, and stability. In this step, we predicted the secondary structure of our suggested protein using the Gor IV method of the Prabi server which is accessible on (https://npsa-prabi.ibcp.fr/cgi-bin/npsa_automat/) (Sapay et al., 2006). In the next step, the 3D structure of the candidate protein is predicted using the I-TASSER (as ‘Zhang-Server’) server (Yang et al., 2015). This server is accessible at https://zhanglab.ccmb.med.umich.edu/I-TASSER/.

### 2.8. Structure refinement of 3D constructed model of fuspMA of SARS-CoV-2

The protein refining process was performed using GalaxyWeb server which accessible on (http://galaxy.seoklab.org/) (HeeShin, 2014) to reduce structural mistakes in the predicted 3D structure of the protein. Also, the quality of the refined model of fuspMA of SARS-CoV-2 was studied using the PROCHECK server (https://servicesn.mbi.ucla.edu/PROCHECK/) (Laskowski et al., 1996).

### 2.9. Molecular dynamics refinement of corrected structural model of fuspMA of SARS-CoV-2

The newly created model of the designed protein was introduced to the molecular dynamics (MD) simulation to obtain an optimized model and to improve our understanding of its physicochemical properties in detail. Simulations and analyses of produced trajectories of fuspMA of SARS-CoV-2 were performed using Gromacs (version 4.5.5) software package (Van Der Spoel et al., 2005). Created topologies of designed protein by I-TASSER server were defined using OPLS-AA force field. The coordinates were placed in separate cubic boxes, solvated by the SPC216 model for the water molecule, and neutralized by the addition of a sufficient number of ions. After all of the indicated steps, the solvated and neutralized structures were minimized by the steepest descent minimization algorithm until the maximum force <1000.0 kJ/mol/nm was reached. The structure was subjected to 100 ps of MD simulations in the canonical (NVT) ensemble to increase the temperature of the systems to 298 K. After 200 ps of NPT-MD equilibration, the final equilibrated structures were used to carry out 50 ns MD simulations. During the simulations, only hydrogen bonds within a distance cutoff of 0.35 nm between the cooperated donor and acceptor with a donor-hydrogen-acceptor angle cutoff of 30° were taken into account.

The particle-mesh Ewald algorithm was used to account for long-range electrostatic interactions (Simmonett et al., 2014). The 50 ns NPT-MD simulation process was performed three times and all of the related represented data is the average value of indicated three times repeated simulations. For visualization of protein structures, we used VMD (Humphrey et al., 1996), UCSF Chimera programs (Pettersen et al., 2004), and ENDscript 2 server-based tools (Robert and Gouet, 2014).

### 2.10. Investigation of interaction of fuspMA of SARSCoV-2 with human MHC I, MHC II, TLR3, and TLR4 by protein-protein docking approach

Interactions of fuspMA of SARS-CoV-2 with human MHC I, MHC II, TLR3, and TLR4 were investigated by proteinprotein docking procedure. Molecular docking study was performed using the ClusPro server in antibody-mode (https://cluspro.org/help.php) (Kozakov et al., 2017) to predict and measurement of the affinity of fuspMA of SARS-CoV-2 structure after 50 ns MD simulation as a ligand to TLR3 (3ULS.pdb) (Luo et al., 2012), TLR4 (4R7N. pdb) (Loyau et al., 2015), MHC I (4UQ3.pdb) (Choo et al., 2014) and MHC II (4GBX.pdb) (Pos et al., 2012) as a receptor (Vajda et al., 2017). The energy of binding for the docked complexes as weighted score was calculated based on the below equation by Cluspro server;


$${\text{E}}_{\text{Energy of binding}}=0.50\text{Erep}+-0.20\text{Eatt}+600\text{Eelec}+0.25\text{EDARS}$$(
Kozakov et al., 2017).

### 2.11. Construction of optimized nucleotide sequence for the designed DNA vaccine

As noticed before, to designing an efficient DNA vaccine, we designed the nucleotide sequence based on the system of the eukaryotic cells. For this purpose, the amino acid sequence of the suggested vaccine was backtranslated into nucleotide sequences based on eukaryotic codon usage using the Jcat server (http://www.jcat.de/) (Grote et al., 2005). Furthermore, signal peptide and Kozak sequence were added into the initial of the DNA sequence to optimize the transcription and gene expression. To this aim, the nucleic sequence of tissue plasminogen activator (TPA) (GenBank: E02360) was obtained from the NCBI database. Furthermore, to make a cleavage between the vaccine sequence and TPA after the expression process, a protease cleavage site has located between the TPA signal peptide and fuspMA. Finally, the constructed sequence has been cloned into a pVax1 eukaryotic vector to use for in vitro expression. After confirming suitable expression of fuspMA of SARS-CoV-2 in eukaryotic cells in cell culture conditions, it could be applied for the in vivo immunization tests.

## 3. Results

### 3.1. Epitope prediction, selection, and estimation of antigenicity, allergenicity, and toxicity of chosen epitopes

To design a multiepitope vaccine, the nucleocapsid protein, membrane glycoprotein, and ORF8 protein were selected from SARS-CoV-2 virus proteins using the NCBI database as indicated above (Figure 1). In the following, each protein sequence is used for B and T-cell epitope prediction using the IEDB server (Vita et al., 2019). The resulted epitopes and their antigenicity, allergenicity, and toxicity are shown in Table 1. Finally, 3 linear B-cell epitopes and 10 T-cell related epitopes were chosen and considered as a vaccine candidate. To construct an effective vaccine, it is necessary to consider three main factors containing; antigenicity level, allergenicity, and toxicity of the proposed amino acid sequence of the vaccine. Accordingly, the selected epitopes studied for these aims and obtained results confirmed the selected ones have shown appropriate antigenic features and lack of allergenic and toxic properties (Table 1). Between the selected epitopes, “DPNFKD” from nucleocapsid protein and “IQYIDIGNY” from ORF 8 have the highest predicted antigenicity (Table 1).

### 3.2. Construction of a peptide sequence containing sequences of selected antigens and adjuvants

**Figure 2:**
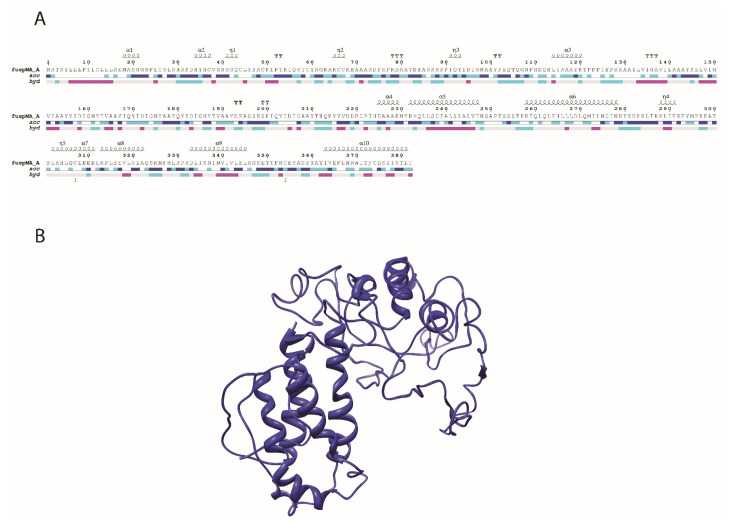
Amino acid sequence, secondary structure (A), and 3D structure (B) of the generated model of fused protein composed of SARS-CoV-2 multiepitope antigens (fusMA of SARS-CoV-2) that created by I-TASSER server based on homology modeling and refined by GalaxyWeb server to reduce structural mistakes in the predicted 3D topologies. fusMA of SARS-CoV-2 is mainly composed of sequences of most potent linear antigens of SRAS-CoV-2, sequences of beta-defensin 1 preproprotein and interleukin-2 as adjuvants, and EAAAK and AAY as linkers.

After selecting of proper epitopes, the primary peptide sequence of the vaccine was designed using linkers and adjuvant sequences. The beta-defensin 1 preproprotein and interleukin-2 were used as adjuvants that connected to epitope sequences by EAAAK linkers. Also, AAY linkers are used to attach the selected epitopes to each other to construct a unique peptide named fuspMA. The amino acid sequence of fuspMA is depicted in Figure 2A.

### 3.3. Physicochemical properties of fuspMA of SARS-CoV-2 as vaccine candidate

**Figure 3:**
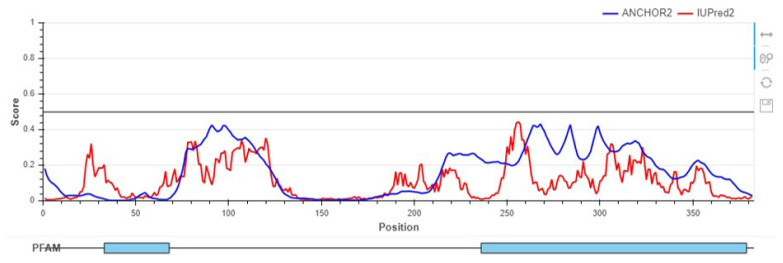
The stability plot of fused protein composed of SARS-CoV-2 multiepitope antigens (fuspMA of SARS-CoV-2) disorders that are drawn by Iupred2a server. Presented data show disordered protein regions using IUPred2 and disordered binding regions using ANCHOR2 of the fuspMA of SARS-CoV-2 refined structure.

As mentioned before, the physicochemical properties of the protein sequence after designing were studied. The obtained results showed, the constructed protein (fuspMA) consists of 382 amino acids with a molecular weight of 42527.46 g/mol. The isoelectric pH and the net charge at pH 7 were predicted at about 5.99 and −4.1, respectively. In the following, the half-life of the protein base on its sequence is estimated about 30 h in mammalian reticulocytes for the in vivo condition. Protein disorder state and possibility of binding of fuspMA to other peptide structures for fuspMA of SARS-CoV-2 that introduces as a vaccine candidate was predicted by Iupred 2.0 designed graph. The designed graph is illustrated in Figure 3. Based on obtained results, the sequence of fuspMA of SARS-CoV-2 has not a great chance to act as an anchor for binding to the registered structures in IUPred2A server (Figure 3).

### 3.4. Prediction of Secondary and 3D structure of fuspMA of SARS-CoV-2

**Figure 4:**
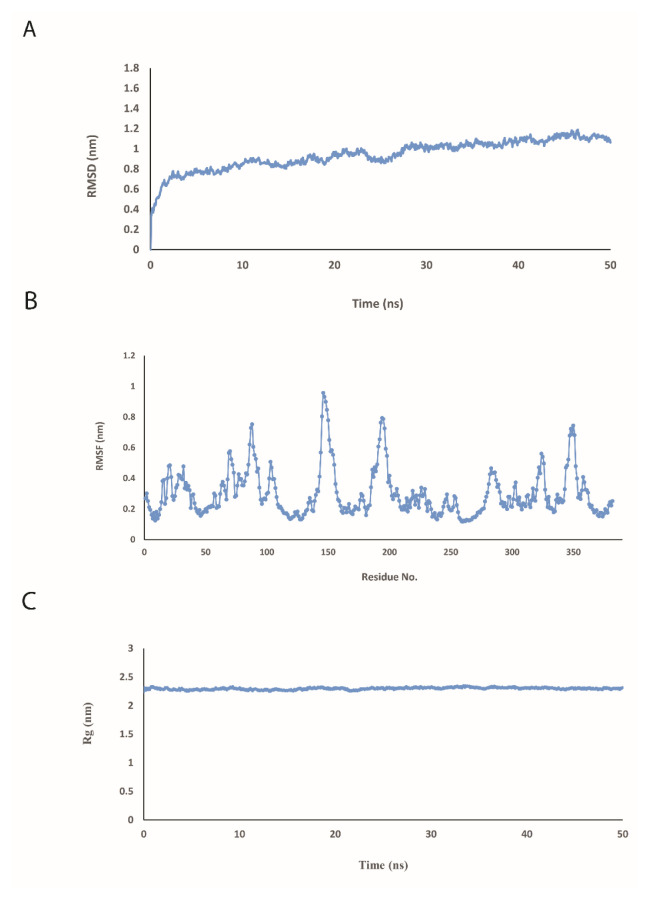
(A) Average of root mean square deviation (RMSD) of Cα of fused protein composed of SARS-CoV-2 multiepitope antigens (fuspMA of SARS-CoV-2). While average of RMSD for fuspMA of SARS-CoV-2 is 0.9395 nm during 50 ns of molecular dynamic simulation, this value is 0.7248 during first 10 ns and 0.9932 for 10 to 50 ns of simulation process. At the 50 ns of MD simulation, RMSD of fuspMA of SARS-CoV-2 was reached to 1.0632 nm in comparison to the original refined 3D model of fuspMA of SARS-CoV-2 that was simulated. (B) Root mean square fluctuations (RMSF) of Cα of residues of fuspMA, (C) and radius of gyration of fuspMA of SARS-CoV-2 (C) during 3 times of 50 ns of molecular dynamic simulation.

**Figure 5:**
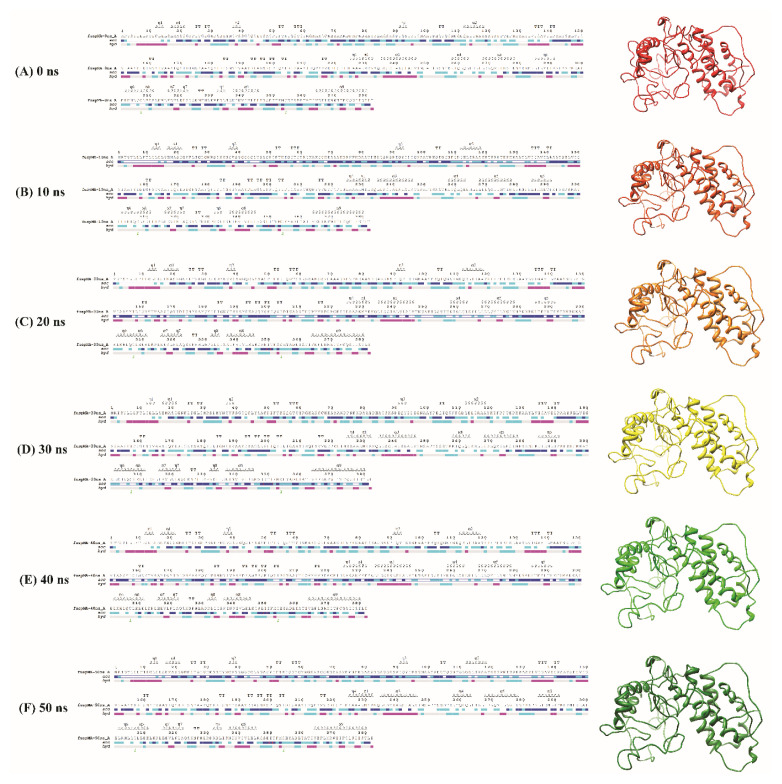
Snapshot of secondary structure and 3D structure of fused protein composed of SARS-CoV-2 multiepitope antigens (fuspMA of SARS-CoV-2) at the beginning of molecular dynamic simulation (A), after 10 ns molecular dynamic simulation (B), after 20 ns molecular dynamic simulation (C), after 30 ns molecular dynamic simulation (D), after 40 ns molecular dynamic simulation (E), after 50 ns molecular dynamic simulation (F).

The secondary and 3D structure of fuspMA of SARSCoV-2 was predicted using I-TASSER server based on the amino acid sequence of the constructed protein and homology modeling of orthologous proteins (Zhang, 2008) and refined by the GalaxyWeb server (Ko et al., 2012) (Figure 2). According to the obtained results, model number 1 with the clash score of 30.5 and RMSD of 0.505 A˚ was the most favorable one (Table 2). The created and refined 3D model of fuspMA by I-TASSER server were minimized and then were simulated. To extend our understanding of fuspMA of SARS-CoV-2, the created 3D model of fuspMA was investigated during 50 ns of MD simulation (Figure 4). As indicated in Figure 4A that depicts root mean square deviation (RMSD) graph of Cα of fuspMA during simulation time, fuspMA of SARS-CoV-2 was reached to its stable conformation after near 10 ns of simulation process and the average of RMSD for the Cα of fuspMA during 50 ns of MD simulation is 0.93951 nm in comparison with the minimized generated structure of fuspMA of SARS-CoV-2 (Figure 4A). Calculated root mean square fluctuations (RMSF) of Cα of residues of fuspMA during 50 ns of simulation process were in ordinary ranges for a stable protein during simulation time (Figure 4B). Furthermore, the average of radius of gyration for fuspMA of SARS-CoV-2 during 50 ns of MD simulation was calculated as 2.2974 nm (Figure 4C). As could be seen in the graph of the radius of gyration for fuspMA of SARS-CoV-2 (Figure 4C), this index did not fluctuate significantly during simulation time that shows the overall structure and the shape of fuspMA of SARS-CoV-2 after reaching to its stable structure was not changed a lot during the simulation process. To investigate the behavior of fuspMA of SARS-CoV-2 during simulation time, secondary and 3D simulated structures were studied every 10 ns (Figure 5). As could be seen in Figure 5 and indicated above, after 10 ns of MD simulation fuspMA of SARS-CoV-2 reached to its stable form, and reported fluctuations could be seen just in the random coil regions of the protein (Figures 4B and 5).

### 3.5. Molecular docking study of interaction of fuspMA of SARS-CoV-2 to immunological response involving receptors

**Figure 6:**
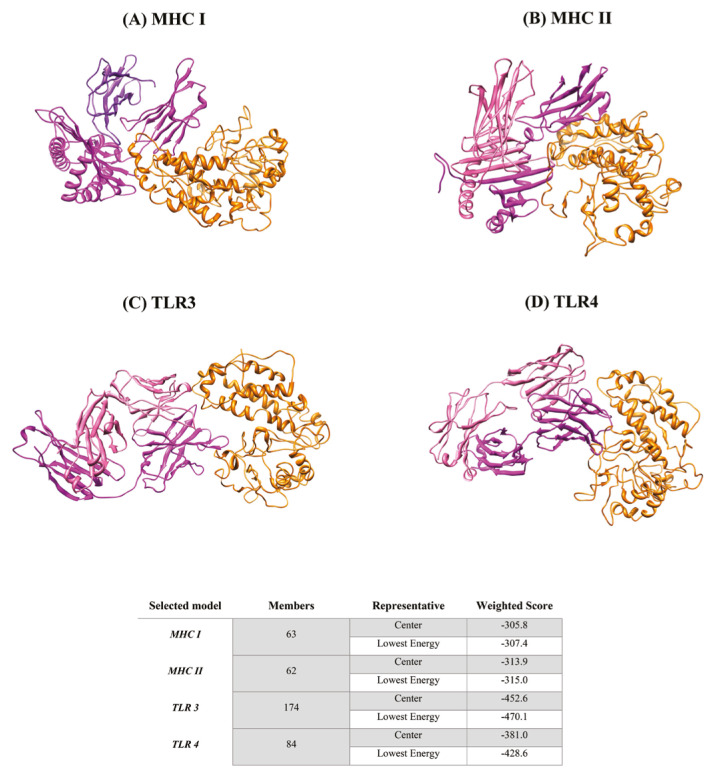
Protein-protein docking of fused protein composed of SARSCoV-2 multiepitope antigens (fuspMA of SARS-CoV-2) (orange red) against human MHC I (purple) (A), MHC II (purple) (B), TLR 3 (purple) (C) and TLR 4 (purple) (D). Weighted scores of docked molecules as center and lowest energies are also reported here.

The molecular docking process was performed using the Cluspro 2.0 protein-protein docking server (https://cluspro.org/help.php) (Vajda et al., 2017) to ensure interactions between receptor molecules containing TLR3, TLR4, MHC I and MHC II and the refined and simulated model of fuspMA as a ligand. The molecular docking process was performed in antibody-mode for each receptor. Docking scores in antibody mode included the center lowest energy of −305.8, −313.9, −452.6, and −381.2 for human MHC I, MHC II, TLR 3, and TLR 4, respectively (Figure 6).

### 3.6. Sequence engineering for designing multiepitope DNA vaccine against COVID-19

**Figure 7:**
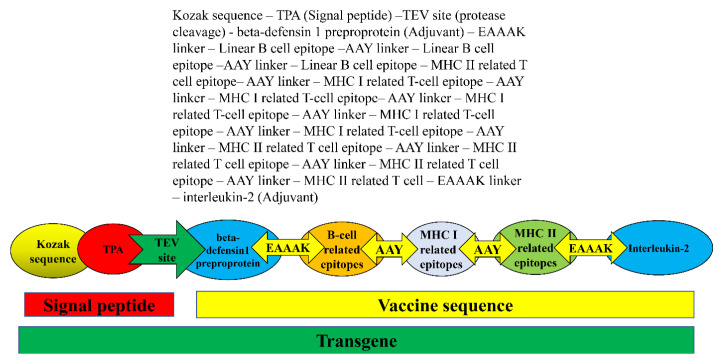
Graphical shape of sequence of the designed multiepitope DNA vaccine against SARS-CoV-2.

**Figure 8:**
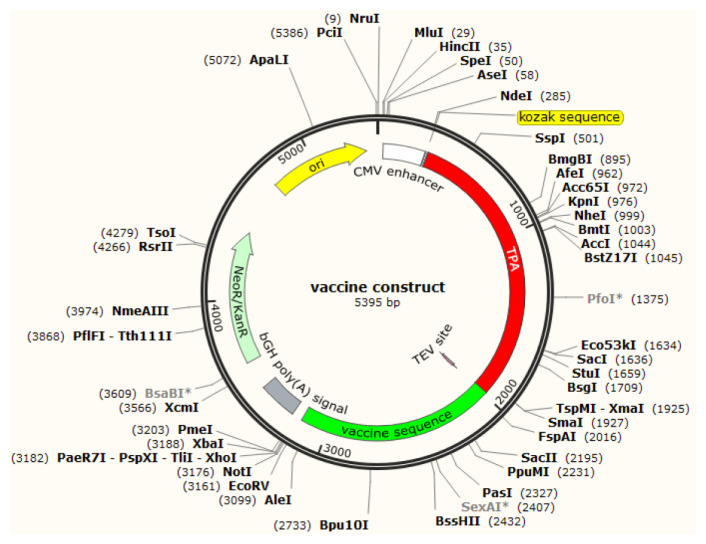
Schematic structure of the designed DNA-vaccine that cloned in pVax1 eukaryotic vector.

To design an effective DNA vaccine some more structural elements should be considered. Firstly, the amino acid sequence of selected epitopes should be backtranslated into a nucleic sequence based on the codon usage algorithm of the target organism. Secondly, signal peptide and Kozak sequence should be added to the first of the nucleic sequence. The graphical view of the final construct of our designed DNA vaccine in order from the N-terminal to C-terminal is illustrated in Figure 7. The final sequence consisted of a Kozak consensus sequence, tissue plasminogen activator (TPA) signal peptide, TEV protease cleavage site, and the vaccine sequence (supplementary 1). In addition, accommodation of the final construct of the proposed multiepitopes DNA-vaccine in pVax1 as eukaryotic expressing vector was demonstrated in Figure 8.

## 4. Discussion

### 4.1. Construction of a chimeric peptide to use for vaccination against COVID-19

Various pathogens cause different diseases in the world and some of them became major health problems for human life. Several outbreaks of viral and bacterial infections were observed previously such as the Q fever outbreak in the Netherlands in 2007, outbreaks of H5N1 influenza in 2003–2004, severe acute respiratory syndrome (SARS) in 2003–2004, and the current global pandemic of the SARS-NEMATI CoV-2 (Zhong et al., 2003; Mase et al., 2005; Karagiannis et al., 2007; Wu et al., 2020).

As mentioned before, the RV method was first used for the development of an effective vaccine against *Meningococcus* bacteria as the causative agent of bacterial meningitis that had no efficacious vaccine before that time (Rappuoli, 2000). This approach was reported notably for various pathogens, so several databases have been created for this object (Rappuoli, 2001). In this strategy of vaccine development, antigenic epitopes of proteins that can stimulate the immune system were selected for further considerations. In the following, chosen epitopes were ordered in an amino acid sequence and connected by linkers. Then, the protein sequence is converted to the DNA sequence that could be synthesized and cloned into suitable vector. Finally, protein expression and purification should be performed to achieve a purified protein to use for animal modeling studies (He et al., 2010). This approach was previously applied for various pathogens and reported in lots of papers (Rappuoli, 2000). For instance, M. Oprea and F. Antohe applied the T-cell epitope as a suggested vaccine against *Staphylococcus aureus*. They selected 9-mer immunogen epitopes of surface-exposed proteins using web databases (Oprea and Antohe, 2013). Furthermore, L. John et al. utilized the RV approach to find vaccine candidates against highly pathogenic species of *Leishmania major* and *Leishmania infantum*. They selected transmembrane proteins from the total proteome of the targets and considered them for further investigations. Finally, they reported 19 epitopes for their recommended vaccine against Leishmania (John et al., 2012). In a recent project, during the current pandemic of the human SARS coronavirus 2, M. Enayatkhani and his colleagues have studied in silico design of a novel multiepitope vaccine against the COVID-19 infection using the reverse vaccinology approach (Enayatkhani et al., 2020). In that project, they have selected the nucleocapsid, ORF3a, and membrane protein as target proteins to examine their potential of immunogenicity as B and T-cell epitopes (Enayatkhani et al., 2020). DNA vaccines or plasmid-based vaccines are the other generations of vaccines which were first introduced in 1990. This strategy performs by inducing protein expression upon direct intramuscular injection of plasmid DNA in myocytes (Wolff et al., 1990). These vaccines are a new type of subunit vaccines that allow protein expression in mammalian cells after introduction of plasmid or recombinant viral vectors encoding the selected protective antigens (Ivory and Chadee, 2004). The innate immunity can be activated by recognition of the double-stranded DNA of the plasmid backbone, while adaptive responses are activated by antigen proteins which were cloned into the vector by involving MHC I or MHC II from CD8^+^ and CD4^+^ T cells. Therefore, DNA vaccines can stimulate innate and adaptive immune responses, simultaneously (Ghanem et al., 2013). Due to the global pandemic of COVID-19 disease, efficient DNA vaccines could be approved because of a series of advantages, including the ease of production, safety, simple administration, and genetic stability. Currently, many researchers heavily investigate the design and production of proper DNA vaccines for COVID-19. Trevor R. F. Smith and his research group have designed a DNA vaccine against SARS coronavirus 2. For this purpose, they have selected spike protein as the major surface antigen of coronaviruses and introduced INO-4800 as a potential COVID-19 vaccine candidate (Smith et al., 2020). Today, ZyCoV-D as a DNA plasmid-based COVID-19 vaccine encoding SARA-CoV-2 spike-protein that is developed by Indian pharmaceutical company is approved in India (Dey et al., 2021). Although the DNA vaccination approach could be a cost-effective and fast method for vaccination in massive populations against pathogens, lacking a proper delivery system is still the main problem. To overcome this challenge, Shah and his colleagues have shown that using nanoparticles can be considered a convenient delivery system that is also able to increase the stability of the vaccine (Shah et al., 2014). Based on our previous knowledge about DNA vaccines, most of the DNA vaccines contain almost one gene that encodes a particular antigen protein (Suhrbier, 1997; Smith et al., 2020). In the current study, we apply a novel strategy to design a new DNA vaccine that contains multiple epitopes from the SARS-CoV-2 virus. Here, we have combined the strategies of the RV and DNA vaccine designing processes to achieve a cost-effective, fast, and protective vaccine against the causal agent for the current COVID-19 pandemic. In addition to the above points, designing a vaccine based on different protein antigens could lead to increase the reliability of the vaccine against various mutations that cause proper immunity versus different variants of the virus between vaccinated communities. Many of existed vaccines are not induced reliable immunity against the newly spread variants of SARS-CoV-2 such Omicron coronavirus and this phenomenon leads to emerging another wave of pandemic just after general community vaccination. To this aim, we designed a multiepitope DNA vaccine encoding epitopes of the nucleocapsid, membrane glycoprotein, and ORF8 proteins of the SARS-CoV 2 virus that are optimized for expression in mammalian cells in order to overcome the mutations and make a proper vaccine against this virus (Figures 7 and 8). We have considered and used both B and T-cell epitopes for our designed DNA vaccine that was previously considered and recommended (Florindo et al., 2020). We investigated the expressed chimeric protein (fuspMA of SARS-CoV-2) by the constructed DNA vaccine and showed fuspMA is a stable peptide and has a suitable half-life in the eukaryotic cells, simultaneously. Furthermore, the antigenicity of the encoded peptide sequence was studied by Vaxijen 2.0 server (Doytchinova and Flower, 2007) and considered a probable antigen with a score of 0.93. In the following, other features of the encoded protein, including physicochemical properties, secondary and 3D structure, amino acid compositions, and geometric features have been predicted using online databases and molecular dynamics simulation approaches.

### 4.2. Evaluation of possible induction of an effective immune response by fusMA of SARS-CoV-2 through its interaction with immune system receptors

Finally, the molecular docking study of the encoded protein (fusMA of SARS-CoV-2) with MHC I and II classes, and TLR3 and 4 molecules was performed and obtained results confirmed the potential of fuspMA as an efficient immunogenic vaccine. Nonclassical major histocompatibility class I (HLA class I histocompatibility antigen) molecule in complex with B2M/beta-2-microglobulin involved in immune self-nonself discrimination (Llano et al., 1998). Peptide-bound HLAE-B2M heterotrimeric complex primarily functions as a ligand for natural killer (NK) cell inhibitory receptor KLRD1-KLRC1 (Braud et al., 1998) and through interaction with CD8^+^ cytotoxic T cells, ultimately triggering antimicrobial immune responses consist of secretion of type 1 cytokines (IL-1, IL-8, TNF-alpha, and IFN-gamma), cell-mediated immunity, IgG1, and IgG3 secretion, and inflammation response that all are critical for an efficient antiviral immune response (Hoare et al., 2006; Walters et al., 2018).

Our docking study confirmed the proper interaction of fusMA of SARS-CoV-2 with HLA class I histocompatibility antigen/beta-2-microglobulin that could result in a strong and effective cell-mediated immunity (Figure 6A). MHC II (HLA class II histocompatibility antigen) binds peptides derived from antigens that access the endocytic route of antigen-presenting cells (APC) and presents them on the cell surface for recognition by the CD4 T-cells. The peptide binding cleft accommodates peptides of 10–30 residues. The presented peptides by MHC class II molecules are generated mostly by degradation of proteins that access the endocytic route, where they are processed by lysosomal proteases and other hydrolases. Exogenous antigens that have been endocytosed by the APC are thus readily available for presentation via MHC II molecules, and for this reason, this antigen presentation pathway is usually referred to as exogenous (Lenormand et al., 2012). Our docking study clarified and confirmed the interaction of fusMA with both HLA class I and class II histocompatibility antigen heterodimers (Figure 6). According to our obtained data, fusMA of SARS-CoV-2 showed a greater affinity for HLA class II that is responsible for humoral immunity that is the major key in the vaccination process (Figure 6). Furthermore, we confirmed the interaction of fusMA of SARS-CoV-2 with TLR3 and TLR 4 that are vital for a potent immune response against such kinds of viruses. TLRs (Toll-like receptors) are a key component of innate and adaptive immunity that control host immune response against pathogens through recognition of molecular patterns that are specific to microorganisms (Brikos and O’Neill, 2008). TLR3 is a nucleotide-sensing TLR that is activated by double-stranded RNA as a sign of viral infection. TLR3 acts via the adapter TRIF/TICAM1, leading to NF-kappa-B activation, IRF3 nuclear translocation, cytokine secretion, and the inflammatory response (de Bouteiller et al., 2005; Johnsen et al., 2006). Toll-like receptor 4 (TLR4) cooperates with LY96 and CD14 to mediate the innate immune response to bacterial lipopolysaccharide (LPS) (Tatematsu et al., 2016) and also acts via MYD88, TIRAP, and TRAF6, leading to NF-kappa-B activation, cytokine secretion, and the inflammatory response (Medzhitov et al., 1997; Arbour et al., 2000; Tatematsu et al., 2016).

## 5. Conclusion

New problems could be solved by new kinds of solutions. Today, all aspects of the human modern lifestyle are influenced by the COVID-19 global pandemic, and production of an efficient vaccine or introduction of an effective medical treatment is an urgent requirement. Although, the older generation of vaccines has abundant advantages but is not so efficient against RNA viruses with a high potential of mutation within their genome that make them stronger with new epitopes (Stobart and Moore, 2014). New technologies could open a novel vista to design a pioneer generation of vaccines and medication. DNA vaccines have lots of advantages such as simple and economical preparation, immediate abundant production, and the ability to express multiple epitopes that could be very important for pathogens with high mutation potential like RNA viruses (Liu, 2003). Here, we designed a multiepitope DNA vaccine that expresses various epitopes of nucleocapsid protein, membrane glycoprotein, and ORF8 protein of SARS-CoV-2 and interleukin-2 and beta-defensin 1 preproprotein as an adjuvant. Based on our computational and predictional studies, the expressed protein by this DNA vaccine (fuspMA) is a stable protein with high and acceptable immunogenicity and tolerable allergenicity and toxicity. We assume this strategy for vaccine development can reduce the time and costs of achieving an efficient vaccine against SARS-CoV-2 in comparison to traditional methods. In the end, further experimental in vivo condition is required to assess the capability of the designed DNA vaccine and to evaluate its competency for induction of appropriate and permanent immunity against SARS-CoV-2.

## Supplementary

Supplementary 1 The final sequence of the designed chimeric vaccine against SARS-CoV-2 was consisted of a Kozak consensus sequence, tissue plasminogen activator (TPA) signal peptide, TEV protease cleavage site and the vaccine sequence.

## Figures and Tables

**Table 1:** Selected epitopes and their estimated antigenicity, allergenicity, and toxicity features for the designed multiepitope DNA vaccine against SARS-CoV-2.

*Epitope*	*Related protein*	*Antigenicity*	*Allergenicity*	*Toxicity*
*DPNFKD*	Nucleocapsid protein	2.84	No	No
*RRGPEQTQGNFGDQELIRQGTDYK*	Nucleocapsid protein	0.62	No	No
*RNSTPGSSRGTSPAR*	Nucleocapsid protein	0.75	No	No
*PEQTQGNFGDQELIR*	Nucleocapsid protein	0.63	No	No
*KTFPPTEPKK*	Nucleocapsid protein	0.76	No	No
*LVIGAVILR*	Membrane glycoprotein	1.10	No	No
*SELVIGAVI*	Membrane glycoprotein	0.64	No	No
*DEAGSKSPIQYIDIGN*	ORF 8	0.911	No	No
*YIDIGNYTV*	ORF 8	1.3	No	No
*IQYIDIGNY*	ORF 8	2.09	No	No
*QYIDIGNYTV*	ORF 8	1.49	No	No
*DEAGSKSPIQYIDIG*	ORF 8	1.02	No	No
*TQHQPYVVDDPCPIH*	ORF8	0.67	No	No

**Table 2:** Results of the refinement process of 3D structure of fuspMA that created by I-TASSER server based on homology modeling of orthologous proteins. The GalaxyWeb server suggested 5 models which ordered based on the 3D refine score.

MODEL	GDT-HA	RMSD	MOLPROBITY	CLASH SCORE	POOR PARAMETERS	RAMA FAVORED
**INITIAL**	1.0000	0.000	4.248	191.4	8.6	74.5
**MODEL 1**	0.9169	0.505	2.834	30.5	1.2	75.0
**MODEL 2**	0.9123	0.511	2.748	29.5	0.9	75.0
**MODEL 3**	0.9175	0.502	2.731	27.7	0.6	74.2
**MODEL 4**	0.9130	0.518	2.826	30.1	1.2	75.3
**MODEL 5**	0.9188	0.493	2.805	28.8	1.2	75.5

## Abbreviations

SARS-CoV-2: Severe acute respiratory syndrome coronavirus 2
TPA: tissue plasminogen activator
fuspMA of SARS-CoV-2: fused protein composed of SARS-CoV-2 Multiepitope Antigens
RV vaccinology: reverse vaccinology
MD simulation: molecular dynamic simulation
RMSD: root mean square deviation
RMSF: root mean square fluctuation
RG: radius of gyration
