# Supplementary figures and images for: Development a novel multiepitope DNA vaccine against human SARS coronavirus-2: an immunoinformatic designing study
Source: Turk J Biol. 2022 Jun 23;46(4):263–76. doi: 10.55730/1300-0152.2615 (PMC10388084; doi:10.55730/1300-0152.2615)

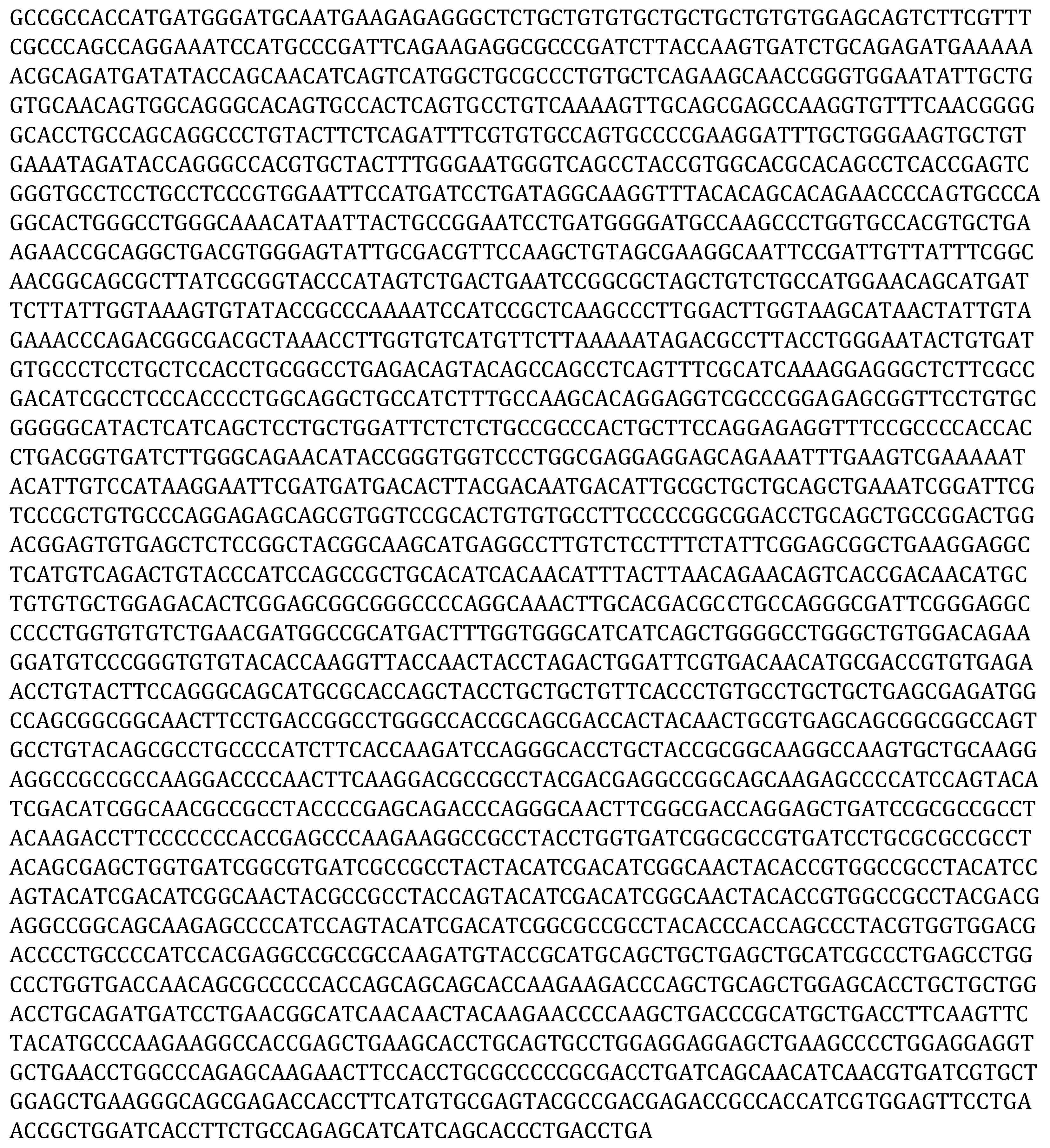

Supplement: Supplementary 1 — The final sequence of the designed chimeric vaccine against SARS-CoV-2 was consisted of a Kozak consensus sequence, tissue plasminogen activator (TPA) signal peptide, TEV protease cleavage site and the vaccine sequence. [file turkjbiol-46-4-263s1.tif]
